# The Americas need to get back on track with immunisation campaigns

**DOI:** 10.1016/j.lana.2022.100353

**Published:** 2022-08-15

**Authors:** The Lancet Regional Health– Americas

Vaccination in the Americas has been considered a model of success for other parts of the world. The Expanded Program on Immunization (EPI), developed by the World Health Assembly and established by PAHO in 1977, has helped to increase the vaccination rate and prevent countless deaths. PAHO´s Revolving Fund for Access to Vaccines allows member states and territories to have access to vaccines at affordable prices. These initiatives enabled great achievements, such as eradicating smallpox in 1971, poliomyelitis in 1994, and rubella and congenital rubella syndrome in 2015, to name a few. August is the National Immunization Awareness month in the USA, and although the Americas should be celebrating their accomplishments, major drops in vaccination coverage in recent years are a cause for concern, accentuated by several new cases of eliminated diseases.

Since 2020, there has been a global drop in vaccination coverage that was further exacerbated by the COVID-19 pandemic. According to PAHO, of 15 million children from the Americas region, 1.4 million do not complete their vaccination cards. Now, 18 countries, including Bolivia, Brazil, El Salvador, and Suriname, reported less than 80% coverage for the first dose of MMR vaccine. Brazil, a country considered an example of vaccination campaigns, with coverage levels achieving almost 100% for many years, has been experiencing a drop for some time. Silveira and colleagues (2021) showed that vaccination coverage in Brazil was reduced in 2019, followed by a bigger decline in 2020 (only about 70% of coverage), with 19% of children missing their scheduled vaccinations. Ecuador experienced similar trends in 2020, with significant reductions for PENTA (17.7%), PV (16.4%), ROTA (12%), and PCV (10.7%) vaccines. With the first case of paralytic poliomyelitis reported in over 10 years in the USA in an unvaccinated young adult, a confirmed case of vaccine-derived poliovirus type 2 (VDPV2), and the presence of poliovirus in environmental samples in New York City, the time to act and increase vaccination rates is now.

According to WHO, one of the top ten threats to global health in 2019 was vaccination hesitancy, which is sadly still true today. Vaccine refusal is not a new event; for example, in 1904, a “Vaccination Riot” took place in Rio de Janeiro, Brazil, for 5 days against the compulsory smallpox vaccination law, which instituted the requirement of immunisation cards for hiring and travelling authorisation, leading to 30 casualties. This is similar to COVID-19, in which vaccination is mandatory in many countries, and many individuals are still refusing to get vaccinated. Misinformation and false claims on vaccines are often available, especially on social media, and are the leading cause of vaccine hesitancy or refusal. Vaccine deniers, or anti-vaxxers, are those who believe that vaccines do not work and could cause diseases. The antivax movement has been increasing for some time now, with serious consequences worldwide. When searching for the term “vaccination” in Google search from the USA, 79% of pages are anti-vaccination. Influenced by the anti-vax movement and misinformation spread through social media, there are also those afraid of the side-effects. Children are highly affected by these movements, as parents are hesitant to follow routine vaccination such as those against human papillomavirus, influenza, and MMR. According to the US CDC, only 70.5% of children born between 2016 and 2017 completed their vaccination routine schedule at age 24 months.

Actions to minimise reluctance to be vaccinated are necessary and need to be re-evaluated and reincorporated, considering that social media influence is here to stay and vaccination refusal is growing in the region. Take measles as an example, a disease considered eliminated in 2016 in the region. Countries such as Canada, Guatemala, Mexico, and Peru reported new measles cases in 2017 and 2018. In 2018, 2801 cases were reported in Brazil, of which 77 were in Indigenous communities; drops in vaccination coverage explain the new cases. In September 2021, the CD59.R13 Resolution entitled “Reinvigorating Immunisation as a Public Good For Universal Health” was released by WHO/PAHO. They propose new strategic goals to improve immunisation programmes in the Americas region. Some of these new goals recommended to member states are to adopt innovative approaches and best practices in immunisation programmes, declare extensive immunisation as a global public good, raise awareness among individuals, protect immunisation-specific budgets, and strengthen governance and leadership of vaccination programmes. There must be combined efforts of PAHO´s programmes and governments to successfully implement each of these goals. Distrust in science and, consequently, vaccines is growing in the region. Government campaigns supporting vaccination, making use of social media to promote science and its impact on population health, and scientists and health-care workers talking directly to the public about the fact that vaccines are genuine life-saving tools are just some of the examples that can be applied immediately.

Immunisation should be a global health-care priority and a human right. Every child and adult should have access to vaccines as they are crucial for preventing and controlling infectious diseases, not only at the individual but also the population level. Vaccines are proven through the history of modern medicine to be a cost-effective health intervention that significantly reduces child mortality. Access to the correct information is crucial to increase public confidence in vaccines and to protect individuals and their communities. A drop in vaccination coverage from a single region could create a pocket of disease outbreak and jeopardise the global effort in eliminating a disease. We call out to each one of the countries in the Americas to boost immunisation awareness earnestly through building and rebuilding trust in science and delivering proper and effective information about vaccines. We also call out to each person and each parent to make a well informed decision to vaccinate themselves or their children.

